# YOLO-Based Simultaneous Target Detection and Classification in Automotive FMCW Radar Systems

**DOI:** 10.3390/s20102897

**Published:** 2020-05-20

**Authors:** Woosuk Kim, Hyunwoong Cho, Jongseok Kim, Byungkwan Kim, Seongwook Lee

**Affiliations:** 1 Machine Learning Lab, AI & SW Research Center, Samsung Advanced Institute of Technology (SAIT), 130, Samsung-ro, Yeongtong-gu, Suwon-si, Gyeonggi-do 16678, Korea; winter.kim@samsung.com (W.K.); hwoong.cho@samsung.com (H.C.); kimbell@samsung.com (J.K.); 2 Department of Radio and Information Communications Engineering, Chungnam National University, 99, Daehak-ro, Yuseong-gu, Daejeon 34134, Korea; byungkwan.kim@cnu.ac.kr; 3 School of Electronics and Information Engineering, Korea Aerospace University, 76, Deogyang-gu, Goyang-si, Gyeonggi-do 10540, Korea

**Keywords:** automotive FMCW radar, target classification, object detection, YOLO

## Abstract

This paper proposes a method to simultaneously detect and classify objects by using a deep learning model, specifically you only look once (YOLO), with pre-processed automotive radar signals. In conventional methods, the detection and classification in automotive radar systems are conducted in two successive stages; however, in the proposed method, the two stages are combined into one. To verify the effectiveness of the proposed method, we applied it to the actual radar data measured using our automotive radar sensor. According to the results, our proposed method can simultaneously detect targets and classify them with over 90% accuracy. In addition, it shows better performance in terms of detection and classification, compared with conventional methods such as density-based spatial clustering of applications with noise or the support vector machine. Moreover, the proposed method especially exhibits better performance when detecting and classifying a vehicle with a long body.

## 1. Introduction

Recently, the automotive market has been in the spotlight in various fields and has been growing rapidly. According to the results obtained in [1], the self-driving car market is expected to be worth $20 billion by 2024 and to grow at a compound annual growth rate of 25.7% from 2016 to 2024. As a result, companies such as Ford, GM, and Audi, which are strong in the existing automobile market, as well as companies such as Google and Samsung, which are not automobile brands currently, have shown interesting in investing in the development of automotive driving. For the complete development of automotive vehicles, several sensors, as well as their well-organization, are required, and the radar sensor is a major sensor for automobiles [2].

In fact, radar has long been used for self-driving vehicles, and its importance has been further emphasized recently. LiDAR, ultrasonics, and video cameras are also considered as competing and complementing technologies in vehicular surround sensing and surveillance. Among the automotive sensors, radar exhibits advantages of robustness and reliability, especially under adverse weather conditions [3]. Self-driving cars can estimate the distance, relative speed, and angle of the detected target through radar, and, furthermore, classify the detected target by using its features such as radar cross-section (RCS) [4,5], phase information [6], and micro-Doppler signature [7,8,9]. In addition, the radar mounted on autonomous vehicles can recognize the driving environment [10,11].

With the development of autonomous driving using radar with deep learning, a few studies have been conducted on radar with artificial neural networks [6,12]. In [13], a fully connected neural network (FCN) was used to replace the traditional radar signal processing, where signals after being subjected to windowing and 2D fast Fourier transform (FFT) were used as training data for FCN with the assistance of a camera. This study showed the feasibility of object detection and 3D target estimation with FCN. In [14,15], the authors presented target classification using radar systems and a convolutional neural network (CNN). After extracting features by using CNN, they used these features to train the support vector machine (SVM) classifier [16]. However, to focus on processing time as well as classification accuracy, we choose the you only look once (YOLO) model among various CNN models. YOLO is a novel model that focuses more on processing time compared with other models [17,18,19]. This model directly trains a network with bounding boxes and class probabilities from full images in one evaluation. As the entire detection pipeline is a single network, it takes less time to obtain the output once an input image is inserted [20].

This paper proposes a simultaneous target detection and classification model that combines an automotive radar system with the YOLO network. First, the target detection results from the range-angle (RA) domain are obtained through radar signal processing, and then, YOLO is trained using the transformed RA domain data. After the learning is completed, we verify the performance of the trained model through the validation data. Moreover, we compare the detection and classification performance of our proposed method with those of the conventional methods used in radar signal processing. Some previous studies have combined the radar system and YOLO network. For example, the authors of [21] showed the performance of the proposed YOLO-range-Doppler (RD) structure, which comprises a stem block, dense block, and YOLO, by using mini-RD data. In addition, the classification performance of YOLO by training it with the radar data measured in the RD domain was proposed in [22]. Both the above-mentioned methods [21,22] dealt with radar data in the RD domain. However, RA data have the advantage of being more intuitive than RD data since the target location information can be expressed more effectively with the RA data. In other words, RA data can be used to obtain the target’s position in a Cartesian coordinate system. Thus, we propose applying the YOLO network to the radar data in the RA domain. In addition, the conventional detection and classification are conducted in two successive stages [5,6], but our proposed method can detect the size of the target while classifying its type. Furthermore, the proposed method has the advantage of detecting and classifying larger objects, compared with the existing method, and can operate in real time.

The remainder of this paper is organized as follows. First, in Section 2, fundamental radar signal processing for estimating the target information is introduced. In Section 3, we present our proposed simultaneous target detection and classification method using YOLO. Then, we evaluate the performance of our proposed method in Section 4. Here, we also introduce our radar system and measurement environments. Finally, we conclude this paper in Section 5.

## 2. Fundamentals of FMCW Radar

### 2.1. How to Estimate Range and Velocity Information with Radar

In general, automotive radar uses a frequency-modulated-continuous-wave (FMCW) radar system with 76–81 GHz bandwidth. A single frame of the FMCW radar comprises a bunch of chirps [23]. Here, we start expressing the signal with a single chirp. The transmission signal for a single chirp of FMCW is expressed as follows:

(1) $$S_{t}\left(\hat{t}\right)=A_{t}\exp\left(j2\pi \left(\left(f_{c}-\frac{B}{2}\right)\hat{t}+\frac{B}{2T_{s}}{\hat{t}}^{2}\right)\right)\;\left(0\leq \hat{t}<T_{s}\right),$$

where $\hat{t}$ indicates the time value in a single chirp and corresponds to the time axis, $A_{t}$ is the amplitude of the transmission signal, $f_{c}$ is the carrier frequency, *B* is the sweep bandwidth, and $T_{s}$ is the sweep time of a single chirp. If this signal is reflected from a target, the corresponding received signal can be expressed as

(2) $$S_{r}\left(\hat{t}\right)=A_{r}\exp\left(j2\pi \left(\left(f_{c}-\frac{B}{2}+f_{D}\right)\left(\hat{t}-t_{d}\right)+\frac{B}{2T_{s}}{\left(\hat{t}-t_{d}\right)}^{2}\right)\right)\;\left(0\leq \hat{t}<T_{s}\right),$$

where $A_{r}$ is the amplitude of the received signal; $f_{d}$ is the Doppler shift frequency, which is induced by a relative velocity, *v*, between the target and the radar; and $t_{d}$ is the round-trip delay, caused by the range, *R*, between the target and the radar.

In general, once the signal is received, it is mixed with the transmitted signal, to be used for signal processing. There are many terms for expressing the mixed signal; however, if we neglect the minor terms, the mixed signal can be expressed as follows:

(3) $$S_{m}\left(\hat{t}\right)\approx A_{m}\exp\left(j2\pi \left(f_{c}\frac{2R}{c}+\left(\frac{2BR}{T_{s}c}-\frac{2f_{c}v}{c}\right)\hat{t}\right)\right)\;\left(0\leq \hat{t}<T_{s}\right),$$

where $A_{m}$ is the amplitude of the mixed signal and *c* is the speed of light. If we consider not a single chirp but the whole frame, Equation (3) can be extended as follows:

(4) $$\begin{array}{c} S_{m}\left(n,\hat{t}\right)\approx A_{m}\exp\left(j2\pi f_{c}\frac{2R}{c}\right)\exp\left(j2\pi \frac{2f_{c}v}{c}T_{s}n\right)\times \exp\left(j2\pi \left(\frac{2BR}{T_{s}c}-\frac{2f_{c}v}{c}\right)\hat{t}\right)\\ (0\leq n<N,\;0\leq \hat{t}<T_{s}), \end{array}$$

where *n* is chirp number of total chirps, *N*, in each frame. If we apply the FFT algorithm with $\hat{t}$ over a single chirp, we can easily find the range information as follows:

(5) $$f_{b}=\frac{2BR}{cT}\;\mathrm{and}\;R=\frac{f_{b}cT}{2B},$$

where $f_{b}$ is a beat frequency, and it is the main frequency component of FFT results with the variable $\hat{t}$. Similar to obtaining *R* from the mixed radar signal, *v* could be derived from the FFT results with the variable *n*, which can be expressed as

(6) $$f_{d}=\frac{2f_{c}v}{c}\;\mathrm{and}\;v=\frac{f_{d}c}{2f_{c}},$$

where $f_{d}$ is the main frequency component of FFT results with the variable *n*.

### 2.2. How to Estimate Angle Information with Radar

If we use the multiple input multiple output antennas on radar system, Equation (4) could be extended as below [24]:

(7) $$\begin{array}{c} S_{m}\left(n,\hat{t},k,p\right)\approx A_{m}\exp\left(j2\pi f_{c}\frac{2R}{c}+j2\pi \frac{2f_{c}v}{c}T_{s}n\right)\times \exp\left(j2\pi \left(\frac{2BR}{T_{s}c}-\frac{2f_{c}v}{c}\right)\hat{t}\right)\\ \times \exp\left(-j2\pi \left(\frac{d_{t}k+d_{r}p}{\lambda }\right)sin\theta \right)\\ (0\leq n<N,\;0\leq \hat{t}<T_{s},\;0\leq k<K,\;0\leq p<P), \end{array}$$

where $d_{t}$ is the distance between adjacent transmit antennas, $d_{r}$ is the distance between adjacent receive antennas, λ is the wavelength of the radar signal, θ is the angle of arrival from the target, *k* is the index of the transmit antennas, *K* is the total number of transmit antennas, *p* is the index of the receive antennas, and *P* is the total number of receive antennas. From Equation (7), we can derive K×P mixed signals from the transmit-receive antenna pairs with the same *n*, $\hat{t}$. If we apply FFT to K×P mixed signals, we can obtain the angle information as follows:

(8) $$\theta =\arcsin\frac{s\lambda }{d_{t}\times K+d_{r}\times P},$$

where *s* is the FFT bin index.

**Figure 1:**
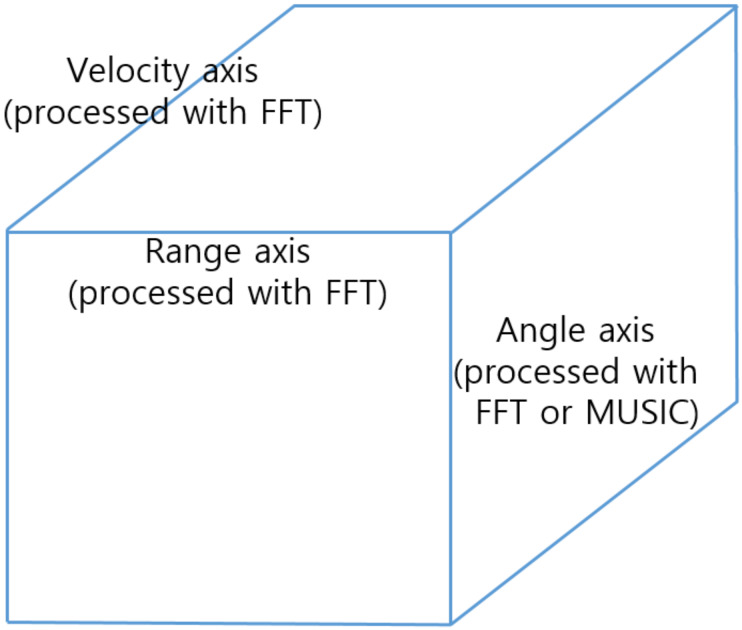
Cube expression of processed radar signal.

However, if the number of antennas is not enough to analyze the degree of arrival, the angle information of the target can be ambiguous or blurred, because of the row angle resolution. To overcome this limitation, we apply the multiple signal classification algorithm to perform experiments with high angle resolution [25,26,27]. Through the process described in Section 2.1 and Section 2.2, we can express the processed radar signal in cubic form, as shown in Figure 1.

## 3. Proposed Simultaneous Detection and Classification Method

### 3.1. Brief Description of YOLO

The YOLO network is a CNN model that uses a single-stage method for object detection and classification. Basically, YOLO considers the bounding box and class probability in an image as a single regression problem, and guesses the type and location of the object by looking at the image only once. At the beginning of the network, the input images are divided into *S* × *S* grid cells. Each grid cell comprises *B* bounding boxes and a confidence score, which represents the object existence probability depending on the intersection over union (IoU). In addition, each grid cell has a conditional class probability, which represents the possibility of whether the object of each class exists. Through network processing, the class-specific confidence score is obtained by multiplying the confidence score and conditional class probability. Finally, YOLO determines the object detection results by comparing the class-specific confidence scores.

The performance of the trained YOLO is expressed in mean average precision (mAP), which is known as a general deep learning performance index. When a new input comes in, YOLO displays the bounding box by estimating the object’s position and class for that input. At this moment, multiple bounding box results may be generated for a single ground truth. Among them, the most leading bounding box can be extracted using non-maximum-suppression (NMS), which is a method for extracting only the bounding box having the highest IoU value. Average precision is the ratio of the positive true among all bounding boxes resulting after NMS on the input, and mAP is the averaged value obtained through all classes, which we have already declared before training.

YOLO has been upgraded steadily, and YOLOv3 is the latest version [28]. YOLOv3 contains 3 × 3 and 1 × 1 convolutional layers and consists of 53 convolutional layers, which is lighter than the other CNN models. Its total loss function (*L*) comprises four terms and it can be expressed as

(9) $$L=L_{cc}+L_{s}+L_{oe}+L_{c},$$

where $L_{cc}$, $L_{s}$, $L_{oe}$, and $L_{c}$ denote the loss caused by the center coordinate error, size error, object existence error, and class error, respectively.

### 3.2. How to Combine Radar Signals with YOLO

**Figure 2:**
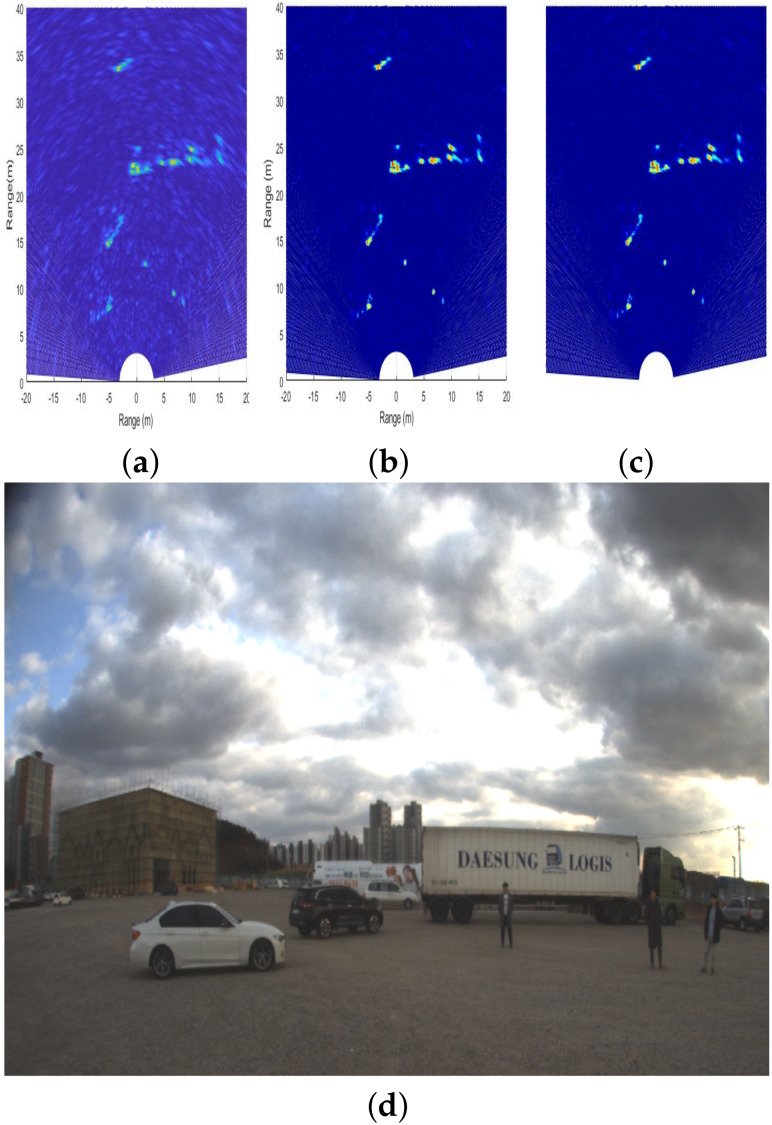
Preprocessing for the input radar image for YOLO network: (**a**) radar image converted into the Cartesian coordinate system; (**b**) radar image after applying the color rule; (**c**) final radar image input to the YOLO network; and (**d**) corresponding camera image.

To apply YOLO to radar signals, it is necessary to express radar signals as images because YOLO network takes images as inputs. When the raw data of the radar is received as shown in Equation (7), the RA domain information can be obtained through the method described in Section 2.1 and Section 2.2. To facilitate labeling of the RA domain information and use 2D images for YOLO training and validation, we take the absolute and mean values on the velocity axis. Then, we plot the RA domain information to a logarithmic scale and convert it into the Cartesian coordinate system. Consequently, we obtain the result with a depth of 3 (i.e., RGB), as shown in Figure 2a.

However, in a general plot method, the color may vary irregularly depending on the signal strength of objects present in the field of view. Therefore, the color rule must be fixed for consistency of training and validation of input images in each scene. As the signal strength changes due to various factors such as the angle between the object and the radar, the RCS characteristic of the object, and abnormal noise, the color rule is fixed by considering the signal strength distribution of the estimated labeled data. The plot is already applied to the logarithmic scale, and the signal intensity distribution is not wide compared to the linear scale plot. As a result, the color rule can be meticulously set for a narrow range of variables. Figure 2b shows the changed plot under the application of a newly defined color rule. Finally, to apply the radar signal image to YOLO, we need to omit the axis. Therefore, as shown in Figure 2c, images with axis expression removed are used for YOLO.

### 3.3. Brief Overview of Proposed Model

**Figure 3:**
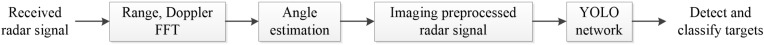
Diagram of proposed model.

Figure 3 shows the flow chart of our proposed model. First, the signal processing described in Section 2.1 is conducted to obtain the range and velocity information from the received radar signal. Then, through the process described in Section 2.2, angle estimation is performed to obtain the cubic data of processed radar signal, as shown in Figure 1. Next, this data cube is imaged through the process described in Section 3.2 to train and exploit the deep learning model. Finally, we can get the detection and classification results after applying the YOLO network with imaged radar signals.

## 4. Detection and Classification Results

### 4.1. Measurement Scenarios

The measurement was conducted on a testing ground with self-produced short-range FMCW radar, which consists of four cascade TI chips, four transmit antennas, and eight receive antennas. The antenna spacings, $d_{t}$ and $d_{r}$, are 0.5λ and field of view of the radar is −60 to 60 degrees. This radar sensor is mounted on the front bumper of the test vehicle. In the measurement, the variables $f_{c}$, *B*, and $T_{s}$ in Equation (1) were set as 77.8 GHz, 1598.4 MHz, and 63.2 us, respectively. In addition, the sampling frequency $f_{s}$ was set as 10 MHz, and the number of the range FFT points was 512. Moreover, one transmission period of the FMCW radar signal was 50 ms, comprised of 4 ms actual transmission time and 46 ms signal processing duration.

In the test field, trailers, cars, and human subjects were used as detectable targets by the automotive FMCW radar system. The detailed information about the targets is shown in Table 1 and Table 2. In the beginning of the measurement, the radar-equipped vehicle was stationary and only a single class moved in each test. The vehicles moved from left to right, right to left, or diagonally, and the pedestrians moved freely within a maximum detection distance of 50 m. Reliable RA data could be obtained until the vehicle was moving at a speed of up to 40 km/h.

Next, data were collected with the radar-equipped vehicle moving toward or away from the stationary targets. In this test scenario, the radar-equipped vehicle moved while keeping the targets within the line of sight of the radar sensor. The data of stationary trailers were mainly collected from their side direction. Finally, we collected the data on human–vehicle mixed targets when the radar-equipped vehicle was moving or stationary. Each frame took 50 ms and all test scenarios were collected for between a minimum of 200 frames and a maximum of 800 frames. In addition, the ground truth information for labeling was obtained using distance-measuring instruments.

The YOLO configuration is listed in Table 3. Most of the configuration follows the recommendation of YOLO developers; however, burn in, max batches, and steps size were modified to fit our model. The data collected from the scenario were labeled, and the labeled data for training or verifying the proposed model are provided in Table 4.

### 4.2. Performance Metric

**Figure 4:**
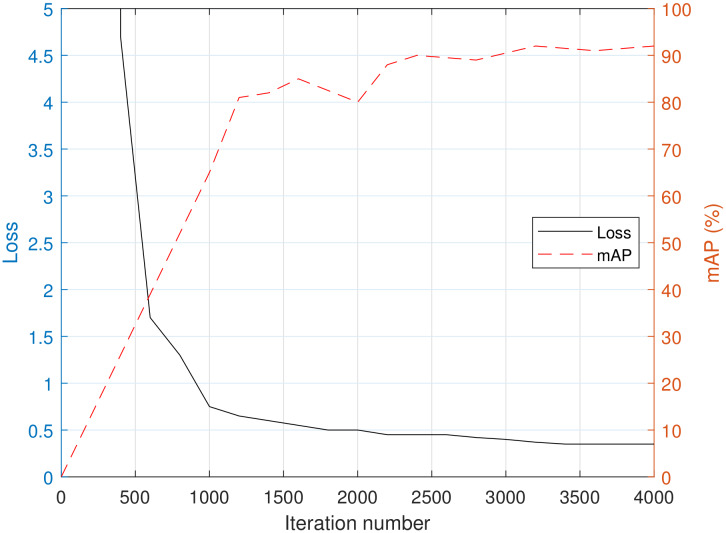
Loss and mAP graph depending on training iteration numbers.

Figure 4 shows the loss function graph on the training iteration axis and evaluated mAP values of the valid data; while learning YOLO with increased training of the model, the loss value decreases. In general, mAP increases at the beginning of training, and then decreases due to overfitting of the deep learning model at a certain number of training iterations. However, in this case, the size of the database is smaller than that of a general deep learning dataset, which seems to have little effect on the performance in the latter part of the training. Checking the performance through valid data after finishing training, the average precision of the trailer is 99.53%, car is 88.82%, and pedestrian is 87.85%. The mAP with 0.5 IoU threshold value is 0.9344 or 93.44%. The inference time per validation image is about 20.16 ms (i.e., around 50 frame per second), which is considered sufficient for automotive radars that require real-time operation.

**Figure 5:**
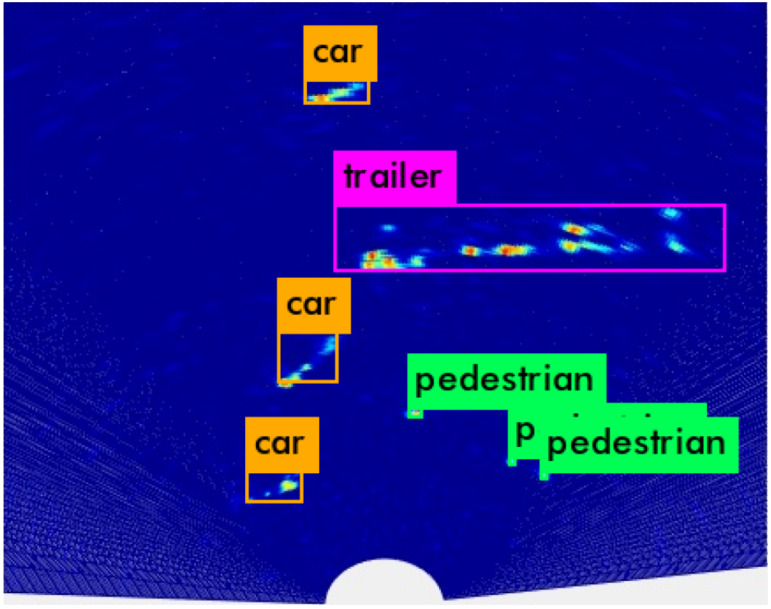
Detection and classification results of our proposed method.

Figure 5 shows the inference result of the proposed network, where a single object is well-recognized with various clusters of multiple detected points. As a result of setting the confidence threshold to 0.25, the true positive is 963, false positive is 56, false negative is 71, and total accuracy is 93.13%, with 1034 ground truth; the average IoU is 74.34%.

#### 4.2.1. Detection

**Figure 6:**
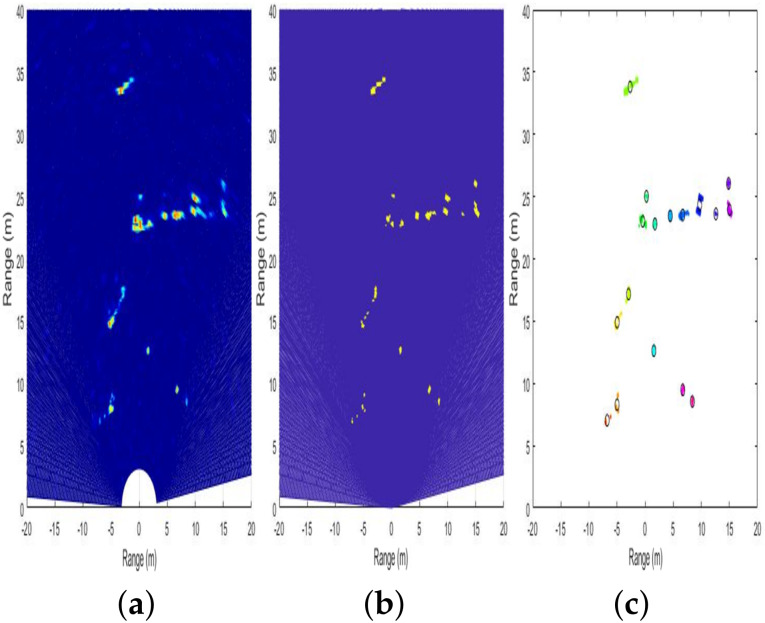
Preprocessing for the input radar signal: (**a**) converted radar image in Cartesian coordinate system; (**b**) radar image after applying OS-CFAR; and (**c**) final clustering result from DBSCAN.

We now compare the conventional method and proposed one from the viewpoint of detection. To compare the performance as fairly as possible, the performance was verified through the following process. Through the pre-processing of radar signals, we could obtain the RA domain information of the detected targets, as shown in Figure 6a. On these RA data, the detection points could be obtained by applying an ordered statistic-constant false alarm rate (OS-CFAR), whose threshold was determined based on the surrounding signal strength [29], as shown in Figure 6b. The detection points identified through CFAR could be grouped with the neighboring detection points through clustering techniques such as density-based spatial clustering of applications with noise (DBSCAN) [30]. However, as shown in Figure 6c, even though the multiple detection points originate from the same object, it cannot be determined as the same cluster from the identical target. Therefore, we propose the following performance indicators to compare the performance with that of the proposed method.

**Figure 7:**
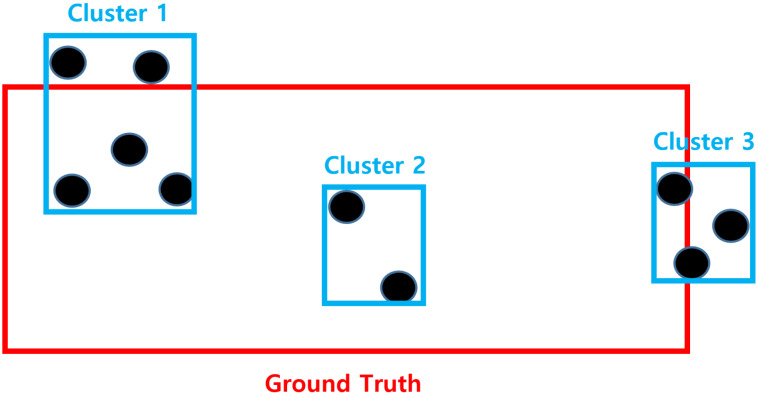
Example for measuring clustering performance.

As shown in Figure 7, each cluster checks the closest ground truth and calculates IoU. If the IoU of cluster is greater than a predefined threshold, it can be considered as the detected cluster of ground truth. In other words, if

(10) $$\frac{\mathrm{IoU}\;\mathrm{between}\;\mathrm{cluster}\;\mathrm{and}\;\mathrm{ground}\;\mathrm{truth}}{\mathrm{Cluster}\;\mathrm{size}}={\mathrm{IoU}}_{\mathrm{Cluster}}>\tau_{\mathrm{IoU}},$$

the cluster is considered to originate from the ground truth. For example, as shown in Figure 7, if the threshold value is 0.5, Clusters 1 and 2 are considered as the detection results of the ground truth. However, Cluster 3 is not the detected result of the ground truth, as the IoU has a very small area. Through this standard, we can derive the cluster sets $Z=[C_{1},\;C_{2},\;\cdots ,\;C_{z}]$ of each ground truth and the total detected area as follows:

(11) $${\mathrm{IoU}}_{\mathrm{total}}=\sum_{z=Z}^{}{\mathrm{IoU}}_{z}.$$

With Equation (11), we can easily obtain the total detected area with the conventional clustering method.

**Figure 8:**
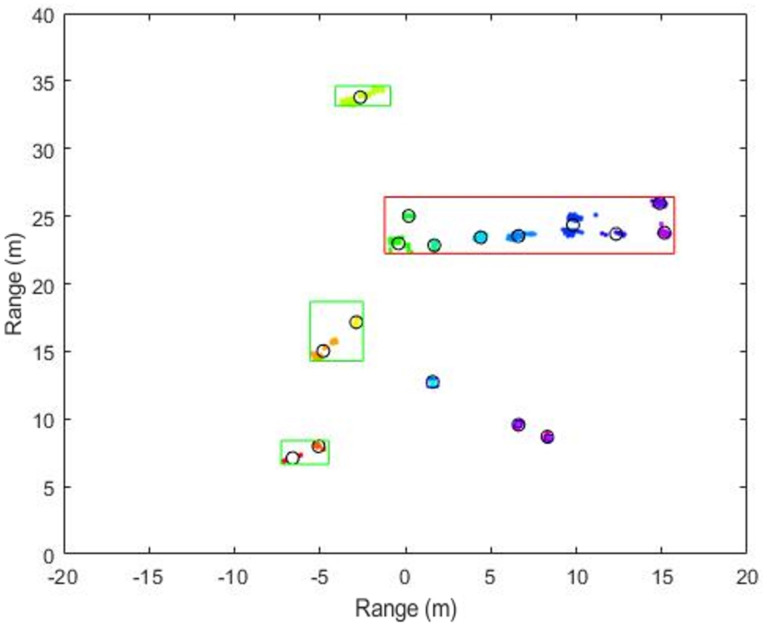
Example of detection performance of the conventional and our proposed methods.

In Figure 8, the red box represents the trailer, the green box represents the car, and the blue box represents the pedestrian of ground truth. Each color represents each cluster and the black circle represents the center position of each cluster. As shown in the figure, under the application of the conventional clustering method, the clusters are recognized as a different group even though they originate from the same object. Especially, in the case of the trailer, as it has larger detected area than other classes, there are more clusters compared to other classes. This is considered to be a phenomenon because, even though it is the same object, the detected points vary depending on the RCS and the angle of arrival of the reflected target area. For example, the car result in Figure 8 comes out as two clusters, as the strong detection points of the wheel parts have characteristic of high RCS.

**Figure 9:**
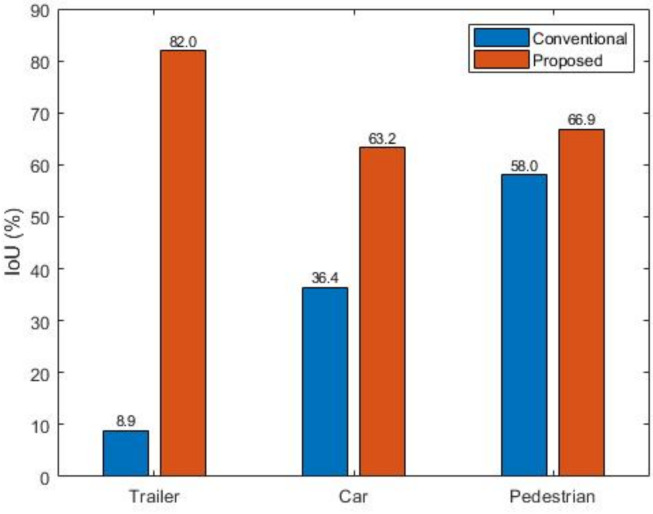
Detection performance comparison between conventional and our proposed methods.

Figure 9 shows the results of the performance indicators mentioned in Section 3. The values for trailers, vehicles, and pedestrians are calculated as 8.9%, 36.4%, and 58.0%, respectively. This indicates that the larger is the object size, the lesser is the detected portion. Compared to the proposed method, which incurs 82.0%, 63.2%, and 66.9% for each class and 74.34% for the average IoU, the proposed method achieves much better performance.

In addition, we compare the computational time between the conventional and proposed methods in Table 5. Unlike the proposed method, the CFAR and clustering are essential processing steps in the conventional method. When the OS-CFAR and DBSCAN were applied, the average processing time was 170.6 ms. Of the total processing time, the processing time of OS-CFAR was about 160 ms, and the processing time of clustering was 10 ms. However, the proposed method is much faster than the existing method because it processes input images at once without such signal processing stage.

#### 4.2.2. Classification

**Figure 10:**
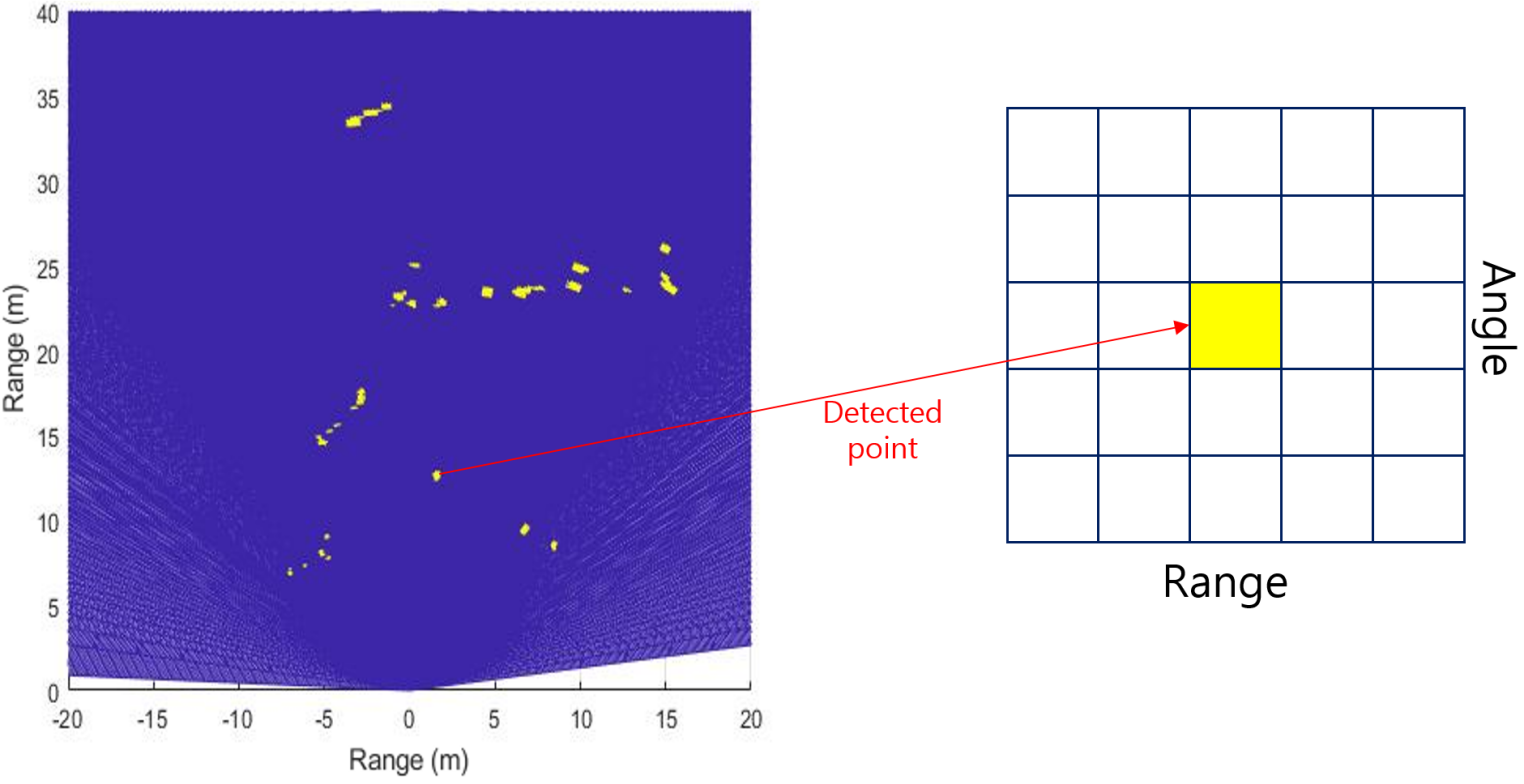
RA data matrix of detected point after CFAR.

After obtaining the detection points by applying CFAR, the labeled data can be used to obtain the coordinate information of real objects, and the class of detected points can be labeled. Once the detected points are classified, we can extract their features using the corresponding information of the RA domain data and compare the classification performance through SVM. The accuracy measured through fifth-fold cross validation, and a linear SVM, which is commonly used in the classification field, is selected. As shown in Figure 10, the 5 × 5-size RA domain matrix data are extracted from each detected point, and seven features (i.e., range, angle, peak value, mean, variance, skewness, and kurtosis) are used for the classification.

**Figure 11:**
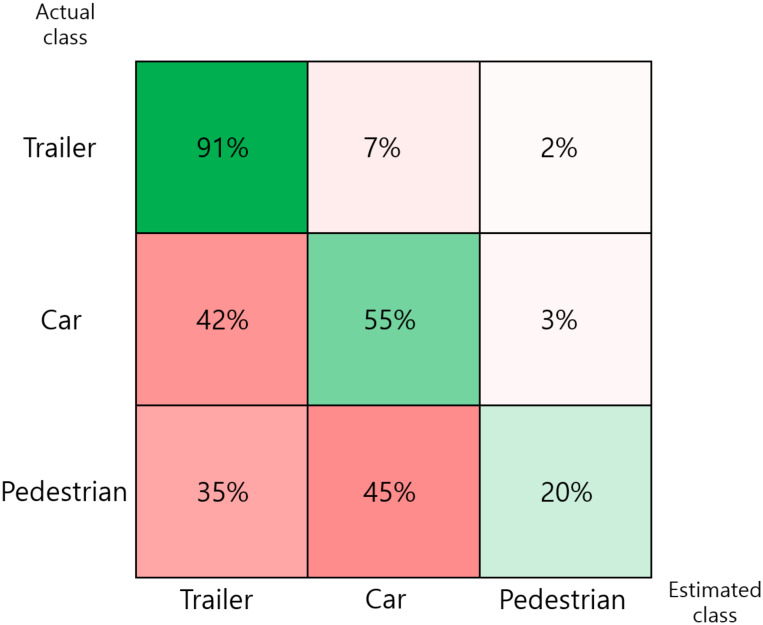
Classification results of the conventional method.

As a result, the overall accuracy of SVM is about 71.8%. Figure 11 shows the accuracy of each class. The trailer shows the best classification performance, while the pedestrian shows the lowest performance. The major reason for this performance gap is the matrix size used for feature extraction. A 5 × 5 matrix is considered to be very large compared to the actual size of the pedestrian. We can reduce the matrix size to 1 × 1 or 3 × 3, but this will decrease the influence of the features, and consequently, decrease the overall accuracy.

Our proposed method achieves an overall classification accuracy of 92.07% with an IoU threshold value of 0.5. This shows much better performance compared to that exhibited by the SVM.

## 5. Conclusions

In this paper, we propose a simultaneous detection and classification method by a using deep learning model, specifically a YOLO network, with preprocessed automotive radar signals. The performance of the proposed method was verified through actual measurement with a four-chip cascaded automotive radar. Compared to conventional detection and classification methods such as DBSCAN and SVM, the proposed method showed improved performance. Unlike the conventional methods, where the detection and classification are conducted successively, we could detect and classify the targets simultaneously through our proposed method. In particular, our proposed method performs better for vehicles with a long body. While the conventional methods recognize one long object as multiple objects, our proposed method exactly recognizes it as one object. This study demonstrates the possibility of applying deep learning algorithms to high resolution radar sensor data, particularly in RA domain. To increase the reliability of the performance of our proposed method, it will be necessary to conduct experiments in various environments.

## Figures and Tables

**Table 1:** Body sizes of vehicles.

	Trailer	Truck	Car 1	Car 2
Length (m)	18	12.5	4.7	4.7
Width (m)	2.5	2.35	1.8	1.8
Type	Dry van	Refrigerator	SUV	Sedan

**Table 2:** Body sizes of four human subjects.

	Subject 1	Subject 2	Subject 3	Subject 4
Height (cm)	175	179	184	185
Weight (kg)	73	83	85	88

**Table 3:** Configuration for YOLO.

Parameter	Value (Unit)
Batch	64
Width	416 (pixels)
Height	416 (pixels)
Channels	3 (R, G, B)
Max batches	4000
Burn in	1000 (batches)
Policy	steps
Learning rate	0.001
Momentum	0.9
Steps	3200, 3600
Decay	0.0005
Scales	0.1, 0.1

**Table 4:** Labeled dataseet for SVM and YOLO.

**SVM**	227,901 detection points
	(125,289 points for trailer, 78,320 points for cars, 24,292 points for pedestrians)
**YOLO**	4028 images with 5837 ground truth
	(1323 ground truth for trailers, 2569 ground truth for cars, 1945 ground truth for pedestrians)

**Table 5:** Computational-time comparison.

	Conventional	Proposed
Processing time (ms)	170.6 ± 10	20.16
	CPU : Intel Xeon Processor E5-2620 v4	
Spec.	GPU : NVIDIA GTX 1080 Ti GDDR5X 11GB * 8	
	RAM : 16GB PC4-19200 ECC-RDIMM * 8	

## Abbreviations

The following abbreviations are used in this manuscript:

CNN	Convolutional neural network
DBSCAN	Density-based spatial clustering of applications with noise
FCN	Fully connected neural network
FFT	Fast Fourier transform
FMCW	Frequency-modulated-continuous-wave
IoU	Intersection over union
mAP	Mean average precision
NMS	Non-maximum-suppression
OS-CFAR	Order statistic-constant false alarm rate
RA	Range-angle
RCS	Radar cross-section
RD	Range-Doppler
SVM	Support vector machine
YOLO	You only look once
